# Motor and non-motor outcome in tremor dominant Parkinson’s disease after MR-guided focused ultrasound thalamotomy

**DOI:** 10.1007/s00415-024-12469-z

**Published:** 2024-05-31

**Authors:** Veronika Purrer, Emily Pohl, Valeri Borger, Hannah Weiland, Henning Boecker, Frederic Carsten Schmeel, Ullrich Wüllner

**Affiliations:** 1 Department of Neurology, University Hospital Bonn, Venusberg-Campus 1, 53127 Bonn, Germany; 2 German Centre of Neurodegenerative Diseases (DZNE), Bonn, Bonn, Germany; 3 Department of Neurosurgery, University Hospital Bonn, Bonn, Germany; 4 Department of Diagnostic and Interventional Radiology, University Hospital Bonn, Bonn, Germany; 5 Department of Neuroradiology, University Hospital Bonn, Bonn, Germany

**Keywords:** Non-motor symptoms, Tremor, Thalamotomy, Focused ultrasound, Parkinson’s disease

## Abstract

**Background and objectives:**

Magnetic Resonance-guided Focused Ultrasound (MRgFUS) is an emerging technique for the treatment of severe, medication-refractory tremor syndromes. We here report motor and non-motor outcomes 6 and 12 months after unilateral MRgFUS thalamotomy in tremor-dominant Parkinson’s disease (tdPD).

**Methods:**

25 patients with tdPD underwent neuropsychological evaluation including standardized questionnaires of disability, quality of life (QoL), mood, anxiety, apathy, sleep disturbances, and cognition at baseline, 6 and 12 months after MRgFUS. Motor outcome was evaluated using the Clinical Rating Scale for Tremor (CRST) and Movement Disorder Society–Unified Parkinson’s Disease Rating Scale (MDS-UPDRS). In addition, side effects and QoL of family caregivers were assessed.

**Results:**

12 months after MRgFUS significant improvements were evident in the tremor subscores. Patients with concomitant rest and postural tremor showed better tremor outcomes compared to patients with predominant rest tremor. There were no differences in the non-motor assessments. No cognitive decline was observed. Side effects were mostly transient (54%) and classified as mild (62%). No changes in the caregivers' QoL could be observed.

**Conclusion:**

We found no changes in mood, anxiety, apathy, sleep, cognition or persistent worsening of gait disturbances after unilateral MRgFUS thalamotomy in tdPD. Concomitant postural tremors responded better to treatment than predominant rest tremors.

**Supplementary Information:**

The online version contains supplementary material available at 10.1007/s00415-024-12469-z.

## Introduction

Parkinson’s disease (PD) is characterized clinically by a variety of motor and non-motor symptoms. PD is increasingly considered a heterogeneous syndrome rather than a clear-cut entity, which is evident also with regard to the main clinical subtypes, i.e. tremor dominant (tdPD), akinetic-rigid or postural instability and gait difficulty type [1]. The prevalent tremor in PD is an asymmetric resting tremor suppressed upon movement initiation. This rest tremor is often accompanied by kinetic and/or postural tremor and the underlying tremor generator(s) remain ill-defined [2]. TdPD patients may experience slower disease progression [1], but the tremor as such can be disabling [3] and medical treatment is often ineffective [4]. Thus, stereotactic treatments, in particular deep brain stimulation (DBS) and thalamotomy, have been successfully used in medication-refractory tremors for many years [5]. Herein, common targets include the posteroventral part of the ventrolateral nucleus of the thalamus (VLpv; corresponding to the ventral intermediate nucleus (VIM) in the Schaltenbrand atlas [6]).

The Magnetic Resonance-guided Focused Ultrasound (MRgFUS), which enables interventional neuromodulation by selective thermal lesions of defined structures in the central nervous system without opening the skull has re-awakened the interest in lesional approaches and several studies demonstrated the efficacy and safety of the procedure in tdPD [7–9]. While motor efficacy and side effects such as paraesthesias are observed immediately or within the first few days after treatment, waning effects or effects on cognition and other neuropsychological symptoms might occur subsequently. Previous studies mainly focused on disability, quality of life and cognition, and neuropsychological changes have been less studied extensively [7, 8, 10, 11].

Herein, we analyzed the clinical outcome of 25 tdPD patients treated with MRgFUS after 6 and 12 months with a special emphasis on safety, neuropsychological measurements and quality of life (QoL) in patients and family caregivers. Furthermore, differences in tremor outcomes in patients with different tremor manifestations were evaluated.

## Methods

### Patients

From December 2019 to October 2022, we enrolled 25 patients with tdPD according to the IPMDS consensus criteria [2]. The pre-existing diagnosis was confirmed in our outpatient department by two movement disorder neurologists (UW and VP). All patients showed a tremor-dominant subtype classified based on established methods using the Movement Disorder Society–Unified Parkinson’s Disease Rating Scale (MDS-UPDRS) III [12] (see Suppl. Methods S1 and [13]). A tremor-dominant subtype was defined by a tremor/non-tremor ratio equal or greater than 1.15. Tremor had to be moderate to severe (score of ≥ 2 in the dominant hand on the Clinical Rating Scale for Tremor (CRST)[14]) and to affect daily activities and/or QoL (score > 2 in the disability suspicion of the CRST or ≥ 30% self-rated reduction of QoL caused by the tremor). Reports of at least two previous medication trials (dopaminergic, anticholinergics, amantadine, clozapine, propranolol, budipine) that failed to achieve satisfactory tremor control were required. Current dopaminergic and other tremor-reducing drugs had to be stable for at least 30 days at the timepoint of enrollment and had to be discontinued prior to treatment (at least 12 h overnight). Exclusion criteria involved structural brain damage, epilepsy, coagulopathies, severe cardiac conditions, history of psychiatric disorders or substance abuse, reported cognitive impairment, or a skull density ratio < 0.3. All patients were also evaluated for and informed about DBS. The study was performed according to the Declaration of Helsinki and approved by the local Ethics Committee (314/18). All participants provided written informed consent.

25 patients were included in the primary outcome analysis at 6 months. 6 patients were lost to follow-up at 12 months (2 patients with an initial good tremor effect denied the follow-up because of waning effects, unfortunately, further information on the extent of worsening have not become available, 1 patient with stable tremor effect refused follow-up because of health problems, 3 patients because of travelling restrictions caused by the COVID-19 pandemic).

### Magnetic resonance-guided focused ultrasound thalamotomy

Treatment was performed in a 3-Tesla MRI system (Discovery MR750w, GE Healthcare, Chicago, IL, USA) and the focused ultrasound system (ExAblate 4000 System, InSightec, Haifa, Israel). The VLpv of the thalamus contralateral to the more affected hand was selected as the target. Localization of the VLpv followed standard stereotactic coordinates (on the level of anterior commissure to posterior commissure (AC-PC) line, 14 mm lateral to midline or 11 mm lateral to the third ventricle wall, 25% of the AC-PC line anterior to PC). Adjustment of the target was based on intraoperative clinical evaluation, i.e. the patient’s feedback regarding tremor reduction and possible side effects and the diffusion tensor imaging (DTI)—derived coordinates of the cerebello-thalamic tract (CTT) and selected ‘no-go areas’, the corticospinal tract (CST) and lemniscus medialis (ML), which had been calculated in advance (details in[15]). For further treatment details, we refer to previous descriptions [16, 17].

### Outcomes

Clinical evaluations were conducted by two neurologists (U.W., 28 and V.P., 5 years of experience in movement disorders) directly before the treatment (T0) and 1–3 days after treatment (T1). Follow-up visits were conducted 6 (T2), and 12 months (T3) after MRgFUS.

### Efficacy

For assessment of the motor outcome, the CRST and MDS-UPDRS III. were used. Higher values indicated more severe symptoms. The CRST consists of three parts: tremor severity in different body parts (part A), motor task performance for both hands (part B), and subjective functional disability related to the tremor (part C). Tremor outcome was measured using a hand-specific subscore combining parts A and B of the treated upper extremity (CRST_mod_, ranging from 0 to 28) and calculated using the Weber-Fechner equation[18], which accounts for the logarithmic relationship between tremor rating R and tremor amplitude T = ((T_f_—T_i_)/T_i_ = 10^(α/N)*ΔR^—1). Hereby, T is a function of the change in tremor ratings ΔR, α is a coefficient for the 0–4 scale and equals to 0.5, and N is the number of items included in the scale.

According to the clinical rating of rest and postural tremor of the upper extremity on the CRST part A, patients were divided into the predominant rest tremor group (rest/postural ratio ≥ 1.5; *n* = 11) and the concomitant rest and postural tremor group (rest/postural ratio < 1.5; *n* = 14).

To further analyze the target-effect relationship, we divided patients according to their individual outcome into the higher tremor improvement group with T ≥ 0.7 (*n* = 12) and the lower tremor improvement group with T < 0.7 *n* = 13) according to the CRST_mod_ score after 6 months (T2).

MDS-UPDRS subscales for tremor, rigidity, bradykinesia, and axial symptoms were calculated in total and separately for the treated and untreated side. Details on the subscale calculation are provided in the Supplementary Material. Clinical evaluation was conducted in an off-medication state (minimum of 4-h withdrawal of antiparkinsonian drugs).

To determine changes in tremor-reducing drugs, levodopa equivalent doses (LED) (in milligrams per day) were assessed according to established conversion rates; anticholinergics and propranolol were recorded in addition [19].

### Non-motor outcomes

We assessed changes in disability (CRST part C, MDS-UPDRS I and II, Non-motor Symptoms Questionnaire (NMSQuest)[20]), QoL (Short-Form-36 questionnaire (SF-36)[21], 39-item Parkinson’s Disease Questionnaire (PDQ-39)[22]), mood (Beck Depression Inventory (BDI)[23] and Geriatric Depression Scale (GDS)[24]), “trait” anxiety (State-Trait Anxiety Scale (STAI-T)[25], apathy (Apathy evaluation scale (AES)[26]), sleep disturbances (Epworth Sleepiness Scale (ESS)[27] and REM Sleep Behavior Disorder Screening Questionnaire (RBDSQ)[28]) at baseline, 6 and 12 months and cognition (Montreal Assessment of Cognition (MoCA), at baseline and 12 months.

Relatives were interviewed on the patient’s cognition using the Functional Activities Questionnaire (FAQ)[29]). In addition, caregivers’ QoL were assessed using the Parkinsonism Carers QoL Questionnaire (PQoL Carers)[30].

### Safety

As safety parameters, the incidence and severity of the reported and observed side effects were monitored. Side effects that were only subjective and not clearly related to MRgFUS treatment without impact on daily activities and QoL were classified as mild. Side effects associated with mild or major restrictions in everyday life were recorded as moderate or severe, respectively. A worsening of motor complications was assessed with the MDS-UPDRS IV. As unsteadiness or gait disturbances are common side effects after MRgFUS treatment, we used the scale for the assessment and rating of ataxia (SARA)[31] and extracted the subitems measuring gait (item 1) and stance (item 2) separately.

### Statistical analysis

Data analysis was performed using IBM SPSS Statistics for Windows, version 25 (IBM Corp., Armonk, N.Y., USA). Evaluation of the normal distribution of the data was performed using the Shapiro–Wilk test. Since most of the data were not normally distributed, non-parametric tests were used for further analysis. Friedman’s test was used to assess within-group changes of the test variables among all timepoints. Post hoc analysis with Wilcoxon signed-rank tests with Bonferroni corrections were used for pairwise comparisons. Differences in tremor outcome between patients with concomitant rest and postural tremor or predominant rest tremor subtype and subgroup analysis of side effects were assessed using the Mann–Whitney *U* test. The same test was used to evaluate whether changes on the neuropsychological assessments differ between patients with high and low tremor improvement. The changes in the different scales were calculated by subtracting the baseline value from the value at T3.

Correlations between demographic data and the baseline motor and non-motor test scores were assessed using Spearman’s correlation coefficient *r*.

*P*-values < 0.05 was considered statistically significant.

## Results

### Patients’ demographics

Detailed demographic and clinical characteristics of the 25 patients are given in Table 1.

**Table 1:** Demographic and clinical characteristics of the study participants (*n* = 25)

Characteristic	Value
Age—yr*	63.7 ± 11.3
Male sex—no. (%)	21 (84)
Right-handedness—no. (%)	23 (92)
Age of onset*	58.3 ± 11.7
Disease duration—yr*	5.4 ± 2.6
Dominant hand treated—no. (%)	17 (68)
LED—mg*	567.1 ± 708.4
No. of previous medications received*^,#^	3.9 ± 1.9
Hoehn and Yahr Scale at baseline – no. (%)	
Stage 1	2 (8)
tage 2	13 (52)
Stage 3	9 (36)
Stage 4	1 (4)

^*^Values are means ± SD

^#^Among these: dopaminergic (100%), anticholinergics (40%), amantadine (52%), clozapine (12%), propranolol (40%), budipine (4%)

*LED* = *Levodopa equivalent doses*

The mean age was 63.7 ± 11.3 years (range 35 to 82) and the mean disease duration 5.4 ± 2.6 years (range 1 to 10). 23 (92%) patients were right-handed and in 17 (68%) patients the targeted VLpv was located in the left hemisphere. The mean tremor/non-tremor ratio was 2.0 ± 2.0 (range 1.0 to 10.7). Comparing the more to the less severely affected side on the MDS-UPDRS scale, asymmetric tremor (ratio ≥ 1.5) was found in 22 (88%) patients. 1 patient with the initial diagnosis of essential tremor developed (additional) PD after years.

### MRgFUS thalamotomy

The mean SDR was 0.44 ± 0.09 (range 0.30–0.68) and the median skull surface area was 359.8 ± 25.6 cm^2^ (range 303–411). On average, 8.7 ± 2.9 (range 5–15) sonications and 3.4 ± 1.5 (range 1–6) ablative sonications (peak voxel temperature > 54 °C) were administered. The mean acoustic energy was 8251.8 ± 3154.7 J (range 3050.9–14,179.8) and the mean peak voxel temperature was 58.8 ± 1.8 °C (range 56.3–61.4). On T1-weighted MRI after 6 months, the median lesion volume was 28.6 ± 19.5 mm^3^ (range 10–88).

### Tremor improvement

Table 2 summarizes the motor outcomes after MRgFUS. At all follow-up timepoints, a significant reduction in tremor scores and subscores of the treated side was found, with the most marked reduction at T1. Tremor was found to be reduced by 78% at T1, 59% at T2, and 60% at T3. At T1, a significant reduction in rigidity and bradykinesia of the treated side was also observed but was no longer significant at T2 and T3. There were no significant changes in the subscores of the untreated side and the subscore of the MDS-UPDRS III.

**Table 2:** Motor outcome after MRgFUS

	Timepoint				
	T0 (*n* = 25)	Friedman test value*	T1 (*n* = 25)^#^	T2 (*n* = 25)^#^	T3 (*n* = 19)^#^
CRST Total score	31.5 ± 15.5	χ^2^(3) = 32.82 *p* < 0.001	11.7 ± 8.8 (Z = 2.24, *p* < 0.001)	19.6 ± 10.4 (Z = 0.97, *p* = 0.121)	15.4 ± 7.2 (Z = 1.74, *p* < 0.001)
Treated arm (CRST_mod_)^‡^	13.8 ± 5.9	χ^2^(3) = 39.34 *p* < 0.001	2.6 ± 2.0 (Z = 2.47, *p* < 0.001)	5.8 ± 4.0 (Z = 1.58, *p* = 0.001)	5.1 ± 2.7 (Z = 1.63, *p* = 0.001)
Untreated arm (CRST_mod_)^‡^	6.0 ± 4.9	χ^2^(3) = 0.92 *p* = 0.820	5.8 ± 5.1	6.2 ± 3.8	6.1 ± 5.1
MDS-UPDRS III Total score	35.6 ± 13.4	χ^2^(3) = 26.28 *p* < 0.001	22.6 ± 16.5 (Z = 2.11, *p* < 0.001)	26.2 ± 13.1 (Z = 1.21, *p* = 0.023)	27.4 ± 18.3 (Z = 1.11, *p* = 0.050)
MDS-UPDRS III Tremor Total score	10.6 ± 3.1	χ^2^(3) = 36.05 *p* < 0.001	3.4 ± 3.1 (Z = 2.40, *p* < 0.001)	6.1 ± 3.4 (Z = 1.37, *p* = 0.007)	5.2 ± 2.5 (Z = 1.61, *p* = 0.001)
Treated side	8.1 ± 1.5	χ^2^(3) = 35.35 *p* < 0.001	1.1 ± 1.4 (Z = 2.32, *p* < 0.001)	3.4 ± 3.0 (Z = 1.42, *p* = 0.004)	2.6 ± 2.3 (Z = 1.63, *p* = 0.001)
Untreated side	2.6 ± 2.3	χ^2^(3) = 1.99 *p* = 0.574	2.4 ± 2.7	2.7 ± 2.3	2.5 ± 2.5
MDS-UPDRS III Bradykinesia Total score	10.8 ± 6.0	χ^2^(3) = 8.21 *p* = 0.042	7.8 ± 7.0 (Z = 1.11, *p* = 0.050)	8.4 ± 4.9 (Z = 0.63, *p* = 0.790)	10.1 ± 8.4 (Z = 0.26, *p* = 1.000)
Treated side	7.1 ± 3.3	χ^2^(3) = 18.87 *p* < 0.001	3.7 ± 3.1 (Z = 1.68, *p* < 0.001)	4.5 ± 2.8 (Z = 1.34, *p* = 0.008)	5.3 ± 3.5 (Z = 0.97, *p* = 0.121)
Untreated side	3.7 ± 3.6	χ^2^(3) = 0.62 *p* = 0.892	4.1 ± 4.7	3.9 ± 3.6	4.8 ± 5.3
MDS-UPDRS III Rigidity Total score	3.7 ± 2.1	χ^2^(3) = 17.91 *p* < 0.001	1.8 ± 1.3 (Z = 1.63, *p* = 0.001)	2.6 ± 1.9 (Z = 0.82, *p* = 0.309)	2.8 ± 2.3 (Z = 1.03, *p* = 0.086)
Treated side	2.4 ± 1.3	χ^2^(3) = 31.14 *p* < 0.001	0.6 ± 0.6 (Z = 1.97, *p* < 0.001)	1.4 ± 0.9 (Z = 0.90, *p* = 0.196)	1.4 ± 1.2 (Z = 1.24, *p* = 0.019)
Untreated side	1.3 ± 1.1	χ^2^(3) = 1.85 *p* = 0.605	1.2 ± 1.0	1.3 ± 1.2	1.5 ± 1.3
MDS-UPDRS III Axial Total score	6.7 ± 3.9	χ^2^(3) = 5.32 *p* = 0.150	8.1 ± 6.1	6.2 ± 5.0	6.8 ± 5.8

Values are means ± SD

^*^P values are based on the Friedman Test (*p* < 0.05). Patients with completed 12-months follow-up were included (*n* = 19)

^#^Post hoc analysis was conducted for all follow-up time points compared to baseline using the Wilcoxon signed-rank test and Bonferroni correction for multiple comparisons (*p*’ value < 0 0.017)

^‡^The modified score was derived from the CRST, part A (3 items) and part B (4 items) for the treated and untreated upper extremity (range 0–28)

*MRgFUS* Magnetic Resonance-guided Focused Ultrasound, *T0* Baseline, *T1* 1–3 days post-MRgFUS, *T2* 6 months post-MRgFUS, *T3* 12 months post-MRgFUS, *CRST* Clinical Rating Scale for Tremor, *CRST*_*mod*_ modified score of the Clinical Rating Scale for Tremor, *MDS-UPDRS* Movement Disorder Society–Unified Parkinson’s Disease Rating Scale

Significant correlations were found for age and the MDS-UPDRS III score as well as its subscores for tremor and axial symptoms. Age at onset furthermore correlated with the axial subscore and disease duration with the MDS-UPDRS III total score (Suppl. Table S1.).

**Fig. 1:**
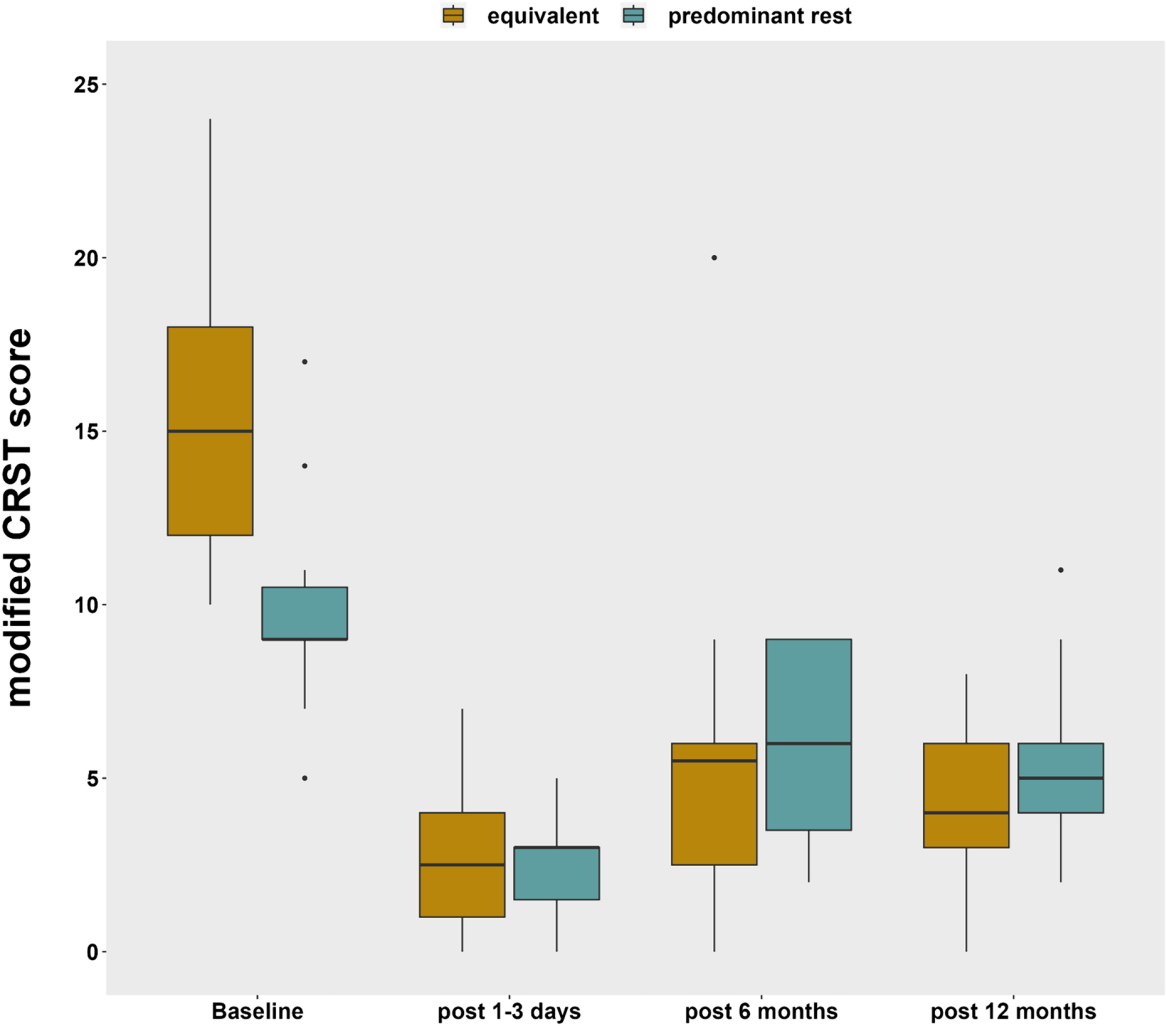
Tremor improvement after MRI-guided focused ultrasound (MRgFUS) thalamotomy in patients with concomitant rest and postural and predominant rest tremor subtype. 12 months post-MRgFUS, significant tremor improvement was observed in the treated extremity—as shown by modified, hand-specific subscores of the Clinical Rating Scale of Tremor (CRST)—in patients with concomitant rest and postural tremor only

Comparing patients with concomitant rest and postural tremor (T1: 87%, T2: 72%, T3: 84%) or predominant rest tremor (T1: 67%, T2: 41%, T3: 38%), patients with concomitant rest and postural tremor showed better tremor outcomes at all follow-up timepoints (T1: p < 0.001, T2: *p* = 0.003, T3: *p* < 0.001). Baseline tremor scores were higher in patients with concomitant rest and postural tremor. Nevertheless, significant tremor improvement 12 months after MRgFUS could be found in patients with concomitant rest and postural tremor only (Fig. 1, Suppl. Table S2).

### Medication

Medical adjustments were made based on parkinsonian symptoms. 12 months after MRgFUS thalamotomy reductions in medications were observed in 6 (32%) patients while an increase was observed in 11 (58%) patients. A significant increase of LED was observed between timepoint T1 and T3 (Z = -1.37, p = 0.007).

### Non-motor outcomes

The non-motor outcomes are given in Table 3. Taken together, only at T1, a significant improvement in the patient’s disability, measured by part C of the CRST, was found which no longer reached significance at the other follow-up visits. No changes were observed in the MDS-UPDRS I and II, and NMSQuest. Furthermore, no differences were observed in the neuropsychological assessments measuring general and PD-specific QoL, mood, anxiety, apathy, and sleep disturbances. Cognitive testing (MoCA) and relatives' interview (FAQ) revealed no cognitive decline at 12 months. The MoCA even showed higher values, corresponding to better cognitive performance.

**Table 3:** Non-motor outcome after MRgFUS

	Timepoint				
	T0 (*n* = 25)	Friedman test value*	T1 (*n* = 25)^#^	T2 (*n* = 25)^#^	T3 (*n* = 19)^#^
CRST, part C (“disability”)	8.3 ± 5.6	χ^2^(3) = 26.26 *p* < 0.001	1.8 ± 2.0 (Z = 2.00, *p* < 0.001)	5.2 ± 4.8 (Z = 0.66, *p* = 0.698)	3.3 ± 3.0 (Z = 1.24, *p* = 0.019)
MDS-UPDRS I (“behaviour and mood”)	9.5 ± 6.8	χ^2^(2) = 8.46 *p* = 0.015		7.4 ± 5.2 (Z = 0.55, *p* = 0.266)	7.2 ± 5.7 (Z = 0.87, p = 0.022)
MDS-UPDRS II (“ADL”)	12.3 ± 6.9	χ^2^(2) = 0.89 *p* = 0.642		12.6 ± 8.2	12.7 ± 8.9
NMSQuest	6.7 ± 4.3	χ^2^(2) = 0.40 *p* = 0.819		6.8 ± 4.1	6.6 ± 3.5
SF-36 PCS	42.2 ± 9.9	χ^2^(2) = 3.44 *p* = 0.179		40.3 ± 10.9	43.3 ± 11.5
MCS	45.5 ± 12.5	χ^2^(2) = 2.33 *p* = 0.311		48.7 ± 9.0	44.6 ± 15.3
PDQ-39 Summary index	20.1 ± 13.0	χ^2^(2) = 4.03 *p* = 0.133		17.6 ± 14.6	20.0 ± 18.2
Mobility	14.7 ± 11.6	χ^2^(2) = 4.51 *p* = 0.105		16.7 ± 16.3	20.7 ± 22.2
Daily activity	33.8 ± 19.5	χ^2^(2) = 5.25 *p* = 0.073		24.5 ± 20.1	26.8 ± 24.7
Emotional wellbeing	20.7 ± 21.7	χ^2^(2) = 0.67 *p* = 0.717		20.0 ± 19.6	21.7 ± 24.1
Stigma	24.3 ± 18.4	χ^2^(2) = 2.94 *p* = 0.230		19.0 ± 15.6	17.4 ± 23.0
Social support	9.7 ± 17.0	χ^2^(2) = 0.84 *p* = 0.657		8.3 ± 14.0	6.6 ± 11.3
Cognition	23.3 ± 21.7	χ^2^(2) = 0.68 *p* = 0.713		18.5 ± 17.4	26.6 ± 23.8
Communication	11.7 ± 14.0	χ^2^(2) = 1.87 *p* = 0.393		14.7 ± 20.2	17.1 ± 24.4
Physical Discomfort	22.7 ± 18.9	χ^2^(2) = 3.58 *p* = 0.167		19.3 ± 20.1	22.8 ± 24.8
BDI	7.8 ± 5.8	χ^2^(2) = 2.32 *p* = 0.313		6.1 ± 4.7	5.9 ± 4.8
GDS	2.3 ± 2.0	χ^2^(2) = 1.89 *p* = 0.390		3.0 ± 2.8	1.7 ± 2.2
STAI-T	40.2 ± 10.3	χ^2^(2) = 6.21 *p* = 0.045		37.3 ± 8.2 (Z = 0.76, *p* = 0.056)	39.3 ± 13.0 (Z = 0.42, *p* = 0.583)
AES	58.7 ± 7.5	χ^2^(2) = 0.61 *p* = 0.738		59.0 ± 7.6	57.3 ± 9.6
ESS	6.5 ± 4.3	χ^2^(2) = 2.55 *p* = 0.219		6.9 ± 4.3	5.7 ± 3.2
RBDSQ	3.5 ± 2.1	χ^2^(2) = 3.03 *p* = 0.280		2.9 ± 1.9	3.9 ± 2.6
MoCA^‡^	25.7 ± 3.1				26.3 ± 2.6 (Z = -2.25, *p* = 0.024)
FAQ	1.4 ± 2.1	χ^2^(2) = 2.00 *p* = 0.368		2.1 ± 3.0	2.7 ± 4.2
PQoL Carers^⁑^	18.4 ± 16.3	χ^2^(2) = 0.63 *p* = 0.729		22.4 ± 21.0	23.1 ± 25.6

Values are means ± SD

^*^P values are based on the Friedman Test (*p* < 0.05). Patients with completed 12-months follow-up were included (*n* = 19)

^#^Post hoc analysis was conducted for all follow-up time points compared to baseline using the Wilcoxon signed-rank test and Bonferroni correction for multiple comparisons (*p*’ value < 0 0.017)

^‡^The Wilcoxon signed-rank test was used to compare the baseline and post-12-months score (*p* < 0.05)

^⁑^*n* = 17 were included

*MRgFUS* Magnetic Resonance-guided Focused Ultrasound, *T0* Baseline, *T1* 1–3 days post-MRgFUS, *T2* 6 months post-MRgFUS, *T3* 12 months post-MRgFUS, *CRST* Clinical Rating Scale for Tremor, *MDS-UPDRS* Movement Disorder Society–Unified Parkinson’s Disease Rating Scale, *ADL* Activities of daily living, *NMSQuest* non-motor Symptoms Questionnaire, *SF-36* Short-Form-36 questionnaire, *PCS* Physical Component Scale, *MCS* Mental Component Scale, *PDQ-39* 39-item Parkinson’s Disease Questionnaire, *BDI* Beck Depression Questionnaire, *GDS* Geriatric Depression Scale, *STAIT* State-Trait Anxiety Scale—“trait” anxiety, *AES* Apathy evaluation scale, *ESS* Epworth Sleepiness Scale, *RBDSQ* REM Sleep Behavious Disorder Questionnaire, *MoCA* Montreal Assessment of Cognition, *FAQ* Functional Activities Questionnaire, *PQoL Carers* Parkinsonism Carers Quality of Life Questionnaire

In addition to the patient’s outcome, no changes in the caregivers’ QoL (PQoL Carers) could be observed (Table 3).

The CRST score prior to treatment (T0) correlated significantly with scales measuring disability (CRST, part C, MDS-UPDRS II, NMSQuest, subscore “daily activity” of the PDQ-39), mood (BDI) and sleep disturbances (ESS, RBDSQ). General (SF-36) and PD-specific QoL (total PDQ-39) did not correlate with the CRST. Moreover, higher age correlated significantly with lower scores in the physical component scale of the SF-36 and the subscore “social support” of the PDQ-39. Age of onset had an impact on the subscore “social support” of the PDQ-39. Duration of tremor did not reveal any significant impact on the different test scores or subdomains at baseline (Suppl. Table S3).

Except for the MoCA and FAQ, the neuropsychological scales measuring the same attribute, correlated with each other. The PD-specific QoL (PDQ-39 Summary index) correlated significantly with all disability scales (CRST, part C, MDS-UPDRS I and II, NMSQuest) as well as with depression (BDI, GDS), anxiety (STAIT), apathy (AES), sleep disturbances (ESS, RBDSQ) and cognition (FAQ). The physical component scale (PCS) of the SF-36 correlated negatively with the disability scales MDS-UPDRS I and II and NMSQuest, depressive symptoms (BDI, GDS), anxiety (STAIT), and the RBDSQ. The mental component scale (MCS) were negatively correlated with the scales BDI, STAIT, AES, and FAQ (Suppl. Table S4).

### Safety parameters

Table 4 and Supplementary Table S5 summarize the side effects related to the MRgFUS procedure and during follow-up.

**Table 4:** Side effects after MRgFUS

	Timepoint			
	Total	T1 (*n* = 25)	T2 (*n* = 25)	T3 (*n* = 19)
Paresthesia				
Any region	10	10	4	4
Face, lips, tongue	8	8	3	3
Hand and fingers	2	2	1	1
Gait disturbance				
Any	21	21	7	7
Objective ataxia on examination	16	16	6	6
Subjective unsteadiness	5	5	1	1
Taste disturbance	7	5	5	5
Dysmetria of the contralateral limb	4	4	1	1
Involuntary movements of the contralateral limb	5	0	5	4
Weakness of the contralateral limb	8	8	5	5
Dysarthria	3	3	1	1
Side effects related to stereotactic frame				
Pin-site bleeding	3			
Numbness of the skull	4			
Side effects related to sonication^#^				
Sensation of “heat”, “pressure” or “pain”	14			
“Flipping” sensation	17			
Clinical examination				
SARA Total score^‡^	4.1 ± 3.0	6.5 ± 5.2 (*p* = 0.072)	3.9 ± 3.4 (*p* = 1.000)	4.2 ± 4.2 (*p* = 1.000)
Subitem 1 (“gait”)^‡^	1.0 ± 1.4	2.1 ± 1.8 (*p* = 0.004)	1.1 ± 1.5 (*p* = 1.000)	1.2 ± 1.5 (*p* = 1.000)
Subitem 2 (”stance”)^‡^	0.6 ± 0.7	1.8 ± 1.4 (*p* = 0.010)	0.6 ± 1.0 (*p* = 1.000)	0.6 ± 0.8 (*p* = 1.000)
MDS-UPDRS IV (“motor complications”)^‡^	1.1 ± 2.5	0.5 ± 1.6 (*p* = 1.000)	1.4 ± 2.3 (*p* = 1.000)	1.9 ± 2.7 (*p* = 1.000)

^*^Values are given for 1–3 days (T1), 6 months (T2) and 12 months (T3) after MRgFUS

^#^Side effects related to sonication occurred only during the 10 to 20 s of sonication

^‡^*P* values are based on Friedman test (p < 0.05) and post hoc analysis using the Wilcoxon signed-rank test and Bonferroni correction for multiple comparisons (p’ < 0.017). Patients with completed 12-months follow-up were included (*n* = 19)

*MRgFUS*  Magnetic Resonance-guided Focused Ultrasound, *T1* 1–3 days post-MRgFUS, *T2* 6 months post-MRgFUS, *T3* 12 months post-MRgFUS, *SARA* Scale for the assessment and rating of ataxia

Procedure-related side effects included flipping sensations (68%), sensation of heat, pressure or pain during sonication (56%), transient numbness of the skull (16%), and pin-site bleeding (12%). Side effects related to thalamotomy were gait disturbances (84%), paraesthesias (40%), weakness of the contralateral limb (32%), taste disturbances (28%), reported involuntary movements of the contralateral limb, most likely corresponding to “thalamic” dystonia (20%). dysmetria (16%) or dysarthria (12%). All cases of reported involuntary movements and 2 of the taste disturbances occurred with delay and were reported at the 6 months visit. Gait disturbances could be divided into clear-cut ataxia on examination (76%) and subjective unsteadiness (24%). Paraesthesias mostly involved the face, lips, and tongue (80%), or the fingers of the contralateral hand (20%). The number of side effects and lesion volume at T1 correlated significantly (*r* = 0.509, *p* = 0.009). Furthermore, in patients with more than 2 side effects larger target adjustments were made—mainly in lateral, anterior and inferior direction—but the differences did not reach statistical significance (Suppl. Fig. S1). Further subgroup analyses between patients with and without gait disturbances, paraesthesias or weakness also revealed no significant differences with regard to target adjustments. Lesion volumes at T1 were significantly larger in patients with weakness of the contralateral limb (*Z* = − 2.15, *p* = 0.03), however, this was no longer evident after 6 months.

Overall, most side effects were initially classified as mild (62%), 28% as moderate, and 10% as severe. 54% of the side effects were transient, 32% resolved partially within 12 months, and 14% remained unchanged.

The SARA subitems 1 (“gait”) and 2 (“stance”) showed significantly worsened scores after the treatment (T1), which completely resolved at T2. The MDS-UPDRS IV did not reveal changes in levodopa-related motor complications (Table 4).

## Discussion

Our study summarizes motor and non-motor outcomes 12 months after unilateral MRgFUS thalamotomy in patients with medication-refractory tdPD. A tremor improvement of 60% on the CRST_mod_ of the treated side was present 12 months after unilateral MRgFUS thalamotomy, comparable to previous studies, reporting the effects of MRgFUS in PD patients [7–9, 32, 33].

Bradykinesia and rigidity of the treated side improved initially but this effect faded over time—either because of disease progression or more likely edema regression. Previous studies targeting the VLpv nucleus, reported diminishing effects on rigidity, too [32]. The adjacent anterior (VLa) and posterodorsal part (VLpd) of the ventralolateral nucleus (corresponding to the ventri-oralis anterior (Voa) and posterior (Vop) nucleus in the Schaltenbrand atlas[6]), which receive pallidal projections, are considered to be associated with rigidity[34] and thus might be affected by perilesional edema after VLpv ablation.

The average tremor recurrence rate six months after MRgFUS is estimated at around 11% overall, with a worse outcome in PD patients compared to ET [33, 35]. We thus evaluated differences in tremor outcomes with regard to different tremor manifestations and found that 12 months after MRgFUS, patients with concomitant rest and postural tremor showed tremor improvement of 84% on the CRST subscale of the treated side (CRST_mod_), whereas patients with predominant rest tremor had only 38% tremor improvement. Although tremor in PD is typically described as asymmetric rest tremor, accompanying postural tremor is common, including the more frequently observed “re-emergent” tremor and the less common “pure postural tremor” [36]. Although rest and postural tremor in PD may share pathophysiological mechanisms involving interaction of cerebello-thalamocortical and basal ganglia-thalamocortical circuits (“dimmer-switch-hypothesis”) [37], different mechanistic hypothesis need to be considered. Transcranial magnetic stimulation in ten PD patients ameliorated rest tremor by stimulation of the primary motor cortex but not by stimulation of the cerebellum, whereas postural tremor was reset by both, suggesting an involvement of the cerebello-thalamo-cortical tract (CTT) particularly in postural tremor [38]. More precise delineation of lesion localization with regard to different responses of rest and postural tremor might help to decipher the different pathophysiological mechanism.

No significant reduction of the LED or dose of anticholinergics and propranolol was observed after MRgFUS treatment. Previous studies reported prevention of increased LED or delayed need for levodopa on the one hand[8, 39] or unchanged or even increased LED after MRgFUS thalamotomy [9]. Nevertheless, treatment of other symptoms, such as bradykinesia and rigidity in progressive disease, surely contributed to altered medications.

Gait disturbances and paraesthesias were the most common side effects [32, 40]. Consistent with observations in essential tremor patients [17, 41], we also observed taste disturbances and delayed occurrence of involuntary movements, most likely corresponding to “thalamic” dystonia. Most side effects were mild and improved over time, indicating a consequence of perilesional edema. Recovery in the SARA subitems “gait” and “stance” was evident at the 6-month visit already. Nevertheless, considering that 46% did not fully recover (32% partially recovered), and given the impact of lesion location and target adjustment on the occurrence of side effects, highlights the importance of individualized and precise targeting.

No changes in disability, mood, anxiety, apathy, sleep disturbances or cognition became evident. Previous studies examining the safety of MRgFUS thalamotomy generally used briefer screening batteries to assess changes in disability, mood, or cognition [7–9]. Sperling et al. evaluated non-motor outcomes with an emphasis on cognition in 20 tdPD patients using a comprehensive test battery. They found improvements in QoL and activities of daily living (ADL), but no decline in cognitive function, mood, and behavior 12 months after thalamotomy [10]. Studies on non-motor symptoms following VIM-DBS are also rare, but improvement of ADL, depression, anxiety, and emotional well-being has been reported^42^. Surprisingly, in our study, tremor severity did not correlate significantly with QoL, while scores measuring disability, mood, anxiety, apathy, sleep, and cognition highly correlated with PDQ-39 and SF-36. Sperling et al. reported similar findings and hypothesized that non-motor symptoms might have a greater impact on QoL than tremor severity [10].

Although we used a comprehensive test battery, most of the tests were based on patient’s self-assessment rather than objective clinical testing. Moreover, the MoCA measures global cognitive function and does not refer to specific cognitive domains.

Further limitations of our study are the small sample size as well as the lack of stable medications. As many patients were followed up by their local neurologist in the meantime, there was a lack of meaningful data regarding the reasons for medication adjustments in many cases. While we tried to overcome this disadvantage by conducting the patient’s examination in the off-medication state, this was feasible in the outpatient condition only for a 4-h withdrawal, as many patients had to manage a long-distance journey. These limitations may indeed have a considerable impact on the motor outcome as well. Hence, stable dopaminergic therapies are recommended for future investigations.

In conclusion, our results confirm that unilateral MRgFUS thalamotomy in tdPD is reasonably safe and efficient. Using a comprehensive screening test battery, no declines in mood, anxiety, apathy, sleep or cognition were observed. Furthermore, the SARA scores indicated no persistent worsening of gait disturbances after MRgFUS. Interestingly, the motor outcome tended to be better in patients with concomitant postural tremor.

### Supplementary Information

Below is the link to the electronic supplementary material.

Supplementary file1 (DOCX 80 KB)

## Data Availability

Anonymized data is available on reasonable request from the corresponding author.

## Abbreviations

AC: Anterior commissure
ADL: Activities of daily living
AES: Apathy evaluation scale
BDI: Beck Depression Inventory
C-GIC: Clinical Global Impression of Change-Scale
CRST: Clinical Rating Scale for Tremor
CRSTmod: Modified, hand-specific subscore of the CRST
CST: Corticospinal tract
CTT: Cerebello-thalamic tract
DBS: Deep brain stimulation
DTI: Diffusion tensor imaging
ESS: Epworth Sleepiness Scale
FAQ: Functional Activities Questionnaire
GDS: Geriatric Depression Scale
LED: Levodopa equivalent doses
MCS: Mental Component Scale
MDS-UPDRS: Movement Disorder Society–Unified Parkinson’s Disease Rating Scale
ML: Lemniscus medialis
MoCA: Montreal Assessment of Cognition
MRgFUS: Magnetic Resonance-guided Focused Ultrasound
NMSQuest: Non-motor Symptoms Questionnaire
PC: Posterior commissure
PCS: Physical Component Scale
PD: Parkinson’s Disease
PDQ-39: 39-Item Parkinson’s Disease Questionnaire
P-GIC: Patient Global Impression of Change-Scale
PQoL Carers: Parkinsonism Carers QoL Questionnaire
QoL: Quality of life
RBDSQ: REM Sleep Behavior Disorder Screening Questionnaire
SARA: Scale for the assessment and rating of ataxia
SF-36: Short-Form-36 questionnaire
STAI-T: State-Trait Anxiety Scale—“trait” anxiety
T0: Evaluation 1–2 days prior to MRgFUS
T1: Evaluation 1–3 days after MRgFUS
T2: Evaluation 6 months after MRgFUS
T3: Evaluation 12 months after MRgFUS
Td: Tremor dominant
VIM: Ventral intermediate nucleus
VLa: Anterior part of the ventrololateral nucleus
VLpd: Posterodorsal part of the ventrolateral nucleus
VLpv: Posteroventral part of the ventrolateral nucleus
Voa: Ventri-oralis anterior nucleus
Vop: Ventri-oralis posterior nucleus
