# Supplementary material for: Motor and non-motor outcome in tremor dominant Parkinson’s disease after MR-guided focused ultrasound thalamotomy
Source: J Neurol. 2024 May 31;271(7):3731–42. doi: 10.1007/s00415-024-12469-z (PMC11233288; doi:10.1007/s00415-024-12469-z)
Supplement: Supplementary file 1 — Supplementary file1 (DOCX 80 KB) [file 415_2024_12469_MOESM1_ESM.docx]

**Table of contents**

**Methods S1.** Subscore calculation

**Table S1.** Spearman’s correlations between demographic data and the baseline motor scores

**Table S2.** Tremor outcome after MRgFUS in different tremor types

**Table S3.** Spearman’s correlations between demographic data and the baseline non-motor scores

**Table S4.** Spearman’s correlations between the baseline non-motor scores

**Table S5.** Characteristics of individual side effects after MRgFUS

**Figure S1.** Mean target adjustments in relation to the occurrence of side effects

**Supplementary methods S1.** Subscore calculation

**Clinical Rating Scale for Tremor**

The modified score of the treated upper extremity was derived from the CRST, part A (3 items, rest, postural and kinetic condition of the treated upper extremity) and part B (4 items, drawing A-C and pouring of the treated side). Each item ranged from 0 to 4; the total score ranged from 0 to 28. Higher values indicated more severe symptoms.

The modified score of the untreated upper extremity was derived from the CRST, part A (3 items, rest, postural and kinetic condition of the untreated upper extremity) and part B (4 items, drawing A-C and pouring of the untreated side). Each item ranged from 0 to 4; the total score ranged from 0 to 28. Higher values indicated more severe symptoms.

**Movement Disorder Society–Unified Parkinson’s Disease Rating Scale**

The tremor/non-tremor ratio was calculated as the division of the following sum scores of MDS-UPDRS part III items:

- Tremor sum score = sum of the items 3.15 to 3.17 (objective tremor at rest and action) divided by 9 (number of items included)
- Non-tremor sum score = sum of the items 3.3 to 3.8 (objective rigidity and bradykinesia testing) divided by 15 (number of items included)

The modified score for tremor was calculated as the sum of the following items of the MDS-UPDRS, part III:

- 3.15 upper limb postural tremor (range 0-8)
- 3.16 upper limb kinetic tremor (range 0-8)
- 3.17 upper and lower limb rest tremor (range 0-16)

The total score ranged from 0 to 32. Higher values indicated more severe symptoms.

The modified score for tremor of the treated/untreated side was calculated as the sum of the following items of the MDS-UPDRS, part III:

- 3.15 unilateral assessment of the treated/untreated upper limb postural tremor (range 0-4)
- 3.16 unilateral assessment of the treated/untreated upper limb kinetic tremor (range 0-4)
- 3.17 unilateral assessment of the treated/untreated upper and lower limb rest tremor (range 0-8)

The total score ranged from 0 to 16. Higher values indicated more severe symptoms.

The modified score for bradykinesia was calculated as the sum of the following items of the MDS-UPDRS, part III:

- 3.4 finger tapping task (range 0-8)
- 3.5 hand movement task (range 0-8)
- 3.6 pronation-supination movements of the hand (range 0-8)
- 3.7 toe tapping task (range 0-8)
- 3.8 leg agility task (range 0-8)

The total score ranged from 0 to 40. Higher values indicated more severe symptoms.

The modified score for bradykinesia of the treated/untreated side was calculated as the sum of the following items of the MDS-UPDRS, part III:

- 3.4 unilateral assessment of the finger tapping task (range 0-4)
- 3.5 unilateral assessment of the hand movement task (range 0-4)
- 3.6 unilateral assessment of the pronation-supination movements of the hand (range 0-4)
- 3.7 unilateral assessment of the toe tapping task (range 0-4)
- 3.8 unilateral assessment of the leg agility task (range 0-4)

The total score ranged from 0 to 20. Higher values indicated more severe symptoms.

The modified score for rigidity was calculated as the sum of the following items of the MDS-UPDRS, part III:

- 3.3 upper and lower limb rigidity (range 0-16)

The total score ranged from 0 to 16. Higher values indicated more severe symptoms.

The modified score for rigidity of the treated/untreated side was calculated as the sum of the following items of the MDS-UPDRS, part III:

- 3.3 unilateral assessment of the treated/untreated upper and lower limb rigidity (range 0-8)

The total score ranged from 0 to 8. Higher values indicated more severe symptoms.

**Suppl. Table S1: Spearman’s correlations between demographic data and the baseline motor scores**

|  | **Age** | | **Age of onset** | | **Disease duration** | |
| --- | --- | --- | --- | --- | --- | --- |
|  | **r** | **p-value** | **r** | **p-value** | **r** | **p-value** |
| **CRST Total score** | 0.048 | 0.822 | 0.010 | 0.964 | 0.343 | 0.093 |
| **MDS-UPDRS III Total score** | 0.449* | 0.024 | 0.393 | 0.052 | 0.417* | 0.038 |
| **MDS-UPDRS III Tremor** | 0.435* | 0.030 | 0.361 | 0.076 | 0.357 | 0.080 |
| **MDS-UPDRS III Bradykinesia** | 0.342 | 0.095 | 0.307 | 0.136 | 0.377 | 0.063 |
| **MDS-UPDRS III Rigidity** | 0.306 | 0.137 | 0.262 | 0.207 | 0.305 | 0.138 |
| **MDS-UPDRS III Axial** | 0.528** | 0.008 | 0.493* | 0.014 | 0.227 | 0.287 |
| * p<0.05 **p<0.01  Abbreviations: r = Spearman’s rank correlation coefficient; CRST = Clinical Rating Scale for Tremor; MDS-UPDRS = Movement Disorder Society–Unified Parkinson’s Disease Rating Scale | | | | | | |

**Suppl. Table S2 Tremor outcome after MRgFUS in different tremor types**

| **Motor outcome – equivalent tremor** | | | | | |
| --- | --- | --- | --- | --- | --- |
|  | **Timepoint** | | | | |
|  | **T0 (n=14)** | **Friedman test value*** | **T1 (n=14)** ^#^ | **T2 (n=14)** ^#^ | **T3 (n=9)** ^#^ |
| CRST  Total score | 38.6 ± 16.5 | χ^2^(3)=18.51  p<0.001 | 13.5 ± 10.5  (Z=2.33, p=0.001) | 20.1 ± 12.1  (Z=1.50, p=0.082) | 17.3 ± 9.1  (Z=2.17, p=0.002) |
| Treated arm (CRST_mod_)^‡^ | 16.9 ± 5.8 | χ^2^(3)=19.21  p<0.001 | 2.7 ± 2.3  (Z=2.39, p=0.001) | 5.6 ± 4.9  (Z=1.72, p=0.028) | 4.2 ± 2.4  (Z=1.89, p=0.011) |
| Untreated arm (CRST_mod_)^‡^ | 7.4 ± 5.7 | χ^2^(3)=1.94  p=0.585 | 7.0 ± 5.0 | 6.4 ± 4.5 | 8.1 ± 6.5 |
| MDS-UPDRS III Total score | 35.3 ± 14.1 | χ^2^(3)=13.59  p=0.004 | 23.4 ± 18.4  (Z=2.11, p=0.003) | 25.4 ± 15.1  (Z=1.56, p=0.064) | 31.3 ± 22.6  (Z=1.00, p=0.602) |
| MDS-UPDRS III Tremor  Total score | 11.4 ± 3.1 | χ^2^(3)=19.07  p<0.001 | 4.3 ± 3.3  (Z=2.44, p<0.001) | 5.6 ± 3.3  (Z=1.78, p=0.021) | 5.8 ± 2.2  (Z=1.78, p=0.021) |
| Treated side | 8.5 ± 1.6 | χ^2^(3)=18.85  p<0.001 | 1.5 ± 1.6  (Z=2.11, p=0.003) | 2.8 ± 3.0  (Z=1.89, p=0.011) | 2.0 ± 2.1  (Z=2.00, p=0.006) |
| Untreated side | 2.9 ± 2.3 | χ^2^(3)=0.95  p=0.815 | 2.8 ± 3.0 | 2.9 ± 2.5 | 3.8 ± 2.7 |
| **Motor outcome – predominant rest tremor** | | | | | |
|  | **Timepoint** | | | | |
|  | **T0 (n=11)** | **Friedman test value*** | **T1 (n=11)** ^#^ | **T2 (n=11)** ^#^ | **T3 (n=10)** ^#^ |
| CRST  Total score | 22.4 ± 8.0 | χ^2^(3)=16.33  p<0.001 | 9.4 ± 5.8  (Z=2.15, p=0.001) | 18.9 ± 8.3 (Z=0.50, p=1.000) | 13.6 ± 4.8 (Z=1.35, p=0.116) |
| Treated arm (CRST_mod_)^‡^ | 10.0 ± 3.2 | χ^2^(3)=20.90  p<0.001 | 2.5 ± 1.4  (Z=2.55, p<0.001) | 6.0 ± 2.8  (Z=1.45, p=0.072) | 5.8 ± 2.9  (Z=1.40, p=0.092) |
| Untreated arm (CRST_mod_)^‡^ | 4.3 ± 3.2 | χ^2^(3)=3.93  p=0.269 | 4.2 ± 3.9 | 6.0 ± 3.0 | 4.3 ± 2.5 |
| MDS-UPDRS III Total score | 36.0 ± 13.2 | χ^2^(3)=13.92  p=0.003 | 21.7 ± 14.7  (Z=2.10, p=0.002) | 27.3 ± 10.7  (Z=0.90, p=714) | 23.9 ± 13.7  (Z=1.20, p=0.226) |
| MDS-UPDRS III Tremor  Total score | 9.7 ± 2.9 | χ^2^(3)=18.10  p<0.001 | 2.4 ± 2.5  (Z=2.35, p<0.001) | 6.7 ± 3.5  (Z=1.00, p=0.500) | 4.6 ± 2.8  (Z=1.45, p=0.072) |
| Treated side | 7.5 ± 1.3 | χ^2^(3)=19.88  p<0.001 | 0.5 ± 0.7  (Z=2.50, p<0.001) | 4.2 ± 2.9  (Z=1.00, p=0.500) | 3.2 ± 2.5  (Z=1.30, p=0.146) |
| Untreated side | 2.2 ± 2.3 | χ^2^(3)=1.97  p=0.579 | 1.8 ± 2.2 | 2.5 ± 2.1 | 1.4 ± 1.6 |
| Values are means ± SD.  * P values are based on the Friedman Test (p<0.05). Patients with completed 12-months follow-up were included (n=9/n=10).  ^#^ Post hoc analysis was conducted for all follow-up time points compared to baseline using the Wilcoxon signed-rank test and Bonferroni correction for multiple comparisons (p’ value<0 0.017).  ^‡^ The modified score was derived from the CRST, part A (3 items) and part B (4 items) for the treated and untreated upper extremity (range 0 to 28).  Abbreviations: MRgFUS = Magnetic Resonance-guided Focused Ultrasound; T0 = Baseline; T1 = 1-3 days post-MRgFUS; T2 = 6 months post-MRgFUS; T3 = 12 months post-MRgFUS; CRST = Clinical Rating Scale for Tremor; CRST_mod_ = modified score of the Clinical Rating Scale for Tremor; MDS-UPDRS = Movement Disorder Society–Unified Parkinson’s Disease Rating Scale; | | | | | |

**Suppl. Table S3: Spearman’s correlations between demographic data and the baseline non-motor scores**

|  | **Age** | | **Age of onset** | | **Disease duration** | | **CRST Total score** | |
| --- | --- | --- | --- | --- | --- | --- | --- | --- |
|  | **r** | **p-value** | **r** | **p-value** | **r** | **p-value** | **r** | **p-value** |
| CRST part C | - 0.025 | 0.905 | - 0.044 | 0.836 | 0.253 | 0.222 | 0.855** | <0.001 |
| MDS-UPDRS I | 0.094 | 0.654 | 0.030 | 0.888 | 0.325 | 0.113 | 0.338 | 0.099 |
| MDS-UPDRS II | 0.202 | 0.333 | 0.184 | 0.378 | 0.264 | 0.202 | 0.563** | 0.003 |
| NMSQuest | - 0.019 | 0.930 | - 0.023 | 0.914 | 0.124 | 0.554 | 0.445* | 0.026 |
| SF-36 |  |  |  |  |  |  |  |  |
| PCS | - 0.429* | 0.032 | - 0.370 | 0.069 | - 0.188 | 0.369 | - 0.101 | 0.631 |
| MCS | 0.296 | 0.151 | 0.313 | 0.128 | - 0.114 | 0.587 | - 0.102 | 0.626 |
| PDQ-39 |  |  |  |  |  |  |  |  |
| Summary index | - 0.049 | 0.816 | - 0.089 | 0.671 | 0.162 | 0.438 | 0.239 | 0.250 |
| Mobility | 0.087 | 0.679 | 0.041 | 0.844 | 0.055 | 0.793 | 0.049 | 0.815 |
| Daily activity | 0.201 | 0.335 | 0.192 | 0.357 | 0.090 | 0.668 | 0.460* | 0.021 |
| Emotional wellbeing | - 0.248 | 0.231 | - 0.285 | 0.167 | 0.167 | 0.426 | 0.098 | 0.642 |
| Stigma | - 0.113 | 0.590 | - 0.184 | 0.380 | 0.285 | 0.168 | 0.197 | 0.344 |
| Social support | - 0.408* | 0.043 | - 0.472* | 0.017 | 0.280 | 0.175 | 0.208 | 0.318 |
| Cognition | - 0.031 | 0.883 | - 0.048 | 0.819 | 0.012 | 0.955 | 0.054 | 0.799 |
| Communication | - 0.215 | 0.302 | - 0.222 | 0.287 | 0.221 | 0.288 | 0.315 | 0.125 |
| Physical Discomfort | 0.285 | 0.167 | 0.281 | 0.174 | - 0.017 | 0.936 | 0.110 | 0.601 |
| BDI | - 0.092 | 0.660 | - 0.125 | 0.553 | 0.306 | 0.136 | 0.411* | 0.041 |
| GDS | - 0.196 | 0.348 | - 0.184 | 0.379 | 0.211 | 0.311 | 0.053 | 0.801 |
| STAI-T | - 0.151 | 0.472 | - 0.246 | 0.236 | 0.377 | 0.063 | 0.113 | 0.591 |
| AES | 0.166 | 0.428 | 0.148 | 0.481 | 0.062 | 0.768 | - 0.048 | 0.819 |
| ESS | - 0.303 | 0.141 | - 0.286 | 0.166 | 0.069 | 0.742 | 0.409* | 0.042 |
| RBDSQ | 0.355 | 0.082 | 0.274 | 0.185 | 0.336 | 0.101 | 0.499* | 0.011 |
| MoCA | - 0.295 | 0.152 | - 0.254 | 0.211 | 0.090 | 0.668 | - 0.119 | 0.570 |
| FAQ | - 0.119 | 0.572 | - 0.169 | 0.419 | 0.315 | 0.125 | 0.239 | 0.250 |
| * p<0.05 **p<0.01  Abbreviations: r = Spearman’s rank correlation coefficient; CRST = Clinical Rating Scale for Tremor; MDS-UPDRS = Movement Disorder Society–Unified Parkinson’s Disease Rating Scale; NMSQuest = non-motor Symptoms Questionnaire; SF-36 = Short-Form-36 questionnaire; PCS = Physical Component Scale; MCS = Mental Component Scale; PDQ-39 = 39-item Parkinson’s Disease Questionnaire; BDI = Beck Depression Questionnaire; GDS = Geriatric Depression Scale; STAIT = State-Trait Anxiety Scale – “trait” anxiety; AES = Apathy evaluation scale; ESS = Epworth Sleepiness Scale; RBDSQ = REM Sleep Behavious Disorder Questionnaire; MoCA = Montreal Assessment of Cognition; FAQ = Functional Activities Questionnaire | | | | | | | | |

**Suppl. Table S4: Spearman’s correlations between the baseline non-motor scores**

|  |  | **MDS-UPDRS I** | **MDS-UPDRS II** | **NMS Quest** | **SF-36 PCS** | **SF-36 MCS** | **PDQ-39 SI** | **Mobility** | **Daily activity** | **Emotional wellbeing** | **Stigma** | **Social support** | **Cognition** | **Communication** | **Physical Discomfort** | **BDI** | **GDS** | **STAI-T** | **AES** | **ESS** | **RBDSQ** | **MoCA** | **FAQ** |
| --- | --- | --- | --- | --- | --- | --- | --- | --- | --- | --- | --- | --- | --- | --- | --- | --- | --- | --- | --- | --- | --- | --- | --- |
| **CRST part C** | **r** | 0.432* | 0.574* | 0.521** | - 0.211 | - 0.076 | 0.400* | 0.178 | 0.574** | 0.200 | 0.229 | 0.304 | 0.206 | 0.371 | 0.112 | 0.440* | 0.160 | 0.177 | - 0.110 | 0.556** | 0.383 | -0.247 | 0.407* |
|  | **p** | 0.031 | 0.003 | 0.008 | 0.312 | 0.719 | 0.048 | 0.396 | 0.003 | 0.338 | 0.271 | 0.140 | 0.323 | 0.068 | 0.595 | 0.028 | 0.445 | 0.398 | 0.599 | 0.004 | 0.059 | 0.234 | 0.044 |
| **MDS-UPDRS I** | **r** |  | 0,711** | 0.459* | - 0.670** | - 0.303 | 0.766** | 0.685** | 0.654** | 0.453* | 0.460* | 0.540* | 0.542* | 0.400* | 0.517** | 0.717** | 0.620** | 0.698** | - 0.339 | 0.459* | 0.569** | -0.203 | 0.703** |
|  | **p** |  | <0.001 | 0.021 | <0.001 | 0.141 | <0.001 | <0.001 | <0.001 | 0.023 | 0.021 | 0.005 | 0.005 | 0.048 | 0.008 | <0.001 | <0.001 | <0.001 | 0.097 | 0.021 | 0.003 | 0.331 | <0.001 |
| **MDS-UPDRS II** | **r** |  |  | 0.421* | - 0.656** | - 0.162 | 0.688** | 0.588** | 0.807** | 0.444* | 0.491* | 0.419* | 0.192 | 0.513** | 0.609** | 0.695** | 0.579** | 0.541** | - 0.259 | 0.421* | 0.574** | -0.092 | 0.562** |
|  | **p** |  |  | 0.036 | <0.001 | 0.439 | <0.001 | 0.002 | <0.001 | 0.026 | 0.013 | 0.013 | 0.359 | 0.009 | 0.001 | <0.001 | 0.002 | 0.005 | 0.210 | 0.036 | 0.003 | 0.663 | 0.003 |
| **NMSQuest** | **r** |  |  |  | - 0.420* | - 0.315 | 0.653** | 0.467* | 0.445* | 0.390 | 0.334 | 0.428* | 0.635** | 0.527** | 0.374 | 0.748** | 0.438* | 0.479* | 0.479* | 0.592** | 0.501* | -0.177 | 0.608** |
|  | **p** |  |  |  | 0.037 | 0.125 | <0.001 | 0.019 | 0.026 | 0.054 | 0.103 | 0.033 | <0.001 | 0.007 | 0.066 | <0.001 | 0.029 | 0.015 | 0.015 | 0.002 | 0.011 | 0.396 | 0.001 |
| **SF-36 PCS** | **r** |  |  |  |  | - 0.181 | - 0.582** | - 0.713** | - 0.545** | - 0.314 | - 0.375 | - 0.106 | - 0.195 | - 0.219 | - 0.624** | - 0.478* | - 0.635** | - 0.490* | 0.131 | -0.258 | -0.483* | 0.009 | -0.231 |
|  | **p** |  |  |  |  | 0.387 | 0.002 | <0.001 | 0.005 | 0.127 | 0.064 | 0.615 | 0.350 | 0.292 | <0.001 | 0.016 | <0.001 | 0.013 | 0.533 | 0.213 | 0.014 | 0.968 | 0.266 |
| **SF-36 MCS** | **r** |  |  |  |  |  | - 0.499* | - 0.287 | - 0.205 | - 0.632** | - 0.441* | - 0.577** | - 0.574** | - 0.488* | -0.133 | - 0.415* | 0.155 | - 0.623** | 0.512** | -0.086 | -0.315 | -0.021 | -0.470* |
|  | **p** |  |  |  |  |  | 0.011 | 0.163 | 0.326 | <0.001 | 0.027 | 0.003 | 0.003 | 0.013 | 0.525 | 0.039 | 0.460 | 0.001 | 0.009 | 0.681 | 0.125 | 0.922 | 0.018 |
| **PDQ-39 Summary index** | **r** |  |  |  |  |  |  | 0.844** | 0.699** | 0.812** | 0.701** | 0.687** | 0.696** | 0.611** | 0.707** | 0.758** | 0.565** | 0.799** | - 0.421* | 0.498* | 0.415* | -0.253 | 0.661** |
|  | **p** |  |  |  |  |  |  | <0.001 | <0.001 | <0.001 | <0.001 | <0.001 | <0.001 | 0.001 | <0.001 | <0.001 | 0.003 | <0.001 | 0.036 | 0.011 | 0.039 | 0.223 | 0.000 |
| **Mobility** | **r** |  |  |  |  |  |  |  | 0.612** | 0.658** | 0.588** | 0.493* | 0.448* | 0.416* | 0.718** | 0.650** | 0.600** | 0.670** | - 0.366 | 0.310 | 0.316 | -0.088 | 0.388 |
|  | **p** |  |  |  |  |  |  |  | 0.001 | <0.001 | 0.002 | 0.012 | 0.025 | 0.039 | <0.001 | <0.001 | 0.002 | <0.001 | 0.072 | 0.132 | 0.124 | 0.676 | 0.055 |
| **Daily activity** | **r** |  |  |  |  |  |  |  |  | 0.457* | 0.581** | 0.362 | 0.239 | 0.353 | 0.453* | 0.509** | 0.440* | 0.455* | - 0.092 | 0.398* | 0.455* | -0.264 | 0.561** |
|  | **p** |  |  |  |  |  |  |  |  | 0.002 | 0.002 | 0.076 | 0.249 | 0.084 | 0.023 | 0.009 | 0.028 | 0.022 | 0.661 | 0.049 | 0.022 | 0.203 | 0.004 |
| **Emotional wellbeing** | **r** |  |  |  |  |  |  |  |  |  | 0.589** | 0.637** | 0.495* | 0.695** | 0.444* | 0.669** | 0.477* | 0.783** | - 0.300 | 0.298 | 0.404* | -0.027 | 0.445* |
|  | **p** |  |  |  |  |  |  |  |  |  | 0.002 | <0.001 | 0.012 | <0.001 | 0.026 | <0.001 | 0.016 | <0.001 | 0.146 | 0.149 | 0.045 | 0.898 | 0.026 |
| **Stigma** | **r** |  |  |  |  |  |  |  |  |  |  | 0.594** | 0.375 | 0.518** | 0.362 | 0.440* | 0.468* | 0.463* | - 0.099 | 0.152 | 0.216 | -0.163 | .0397* |
|  | **p** |  |  |  |  |  |  |  |  |  |  | 0.002 | 0.065 | 0.008 | 0.075 | 0.028 | 0.018 | 0.020 | 0.638 | 0.468 | 0.300 | 0.436 | 0.049 |
| **Social support** | **r** |  |  |  |  |  |  |  |  |  |  |  | 0.426* | 0.485* | 0.337 | 0.469* | 0.445* | 0.562** | - 0.459* | 0.181 | 0.075 | -0.269 | 0.718** |
|  | **p** |  |  |  |  |  |  |  |  |  |  |  | 0.034 | 0.014 | 0.100 | 0.018 | 0.026 | 0.003 | 0.021 | 0.387 | 0.721 | 0.193 | 0.000 |
| **Cognition** | **r** |  |  |  |  |  |  |  |  |  |  |  |  | 0.421* | 0.387 | 0.509** | 0.138 | 0.586** | - 0.460* | 0.427* | 0.325 | -0.305 | 0.561** |
|  | **p** |  |  |  |  |  |  |  |  |  |  |  |  | 0.036 | 0.056 | 0.009 | 0.511 | 0.002 | 0.021 | 0.033 | 0.113 | 0.139 | 0.004 |
| **Communication** | **r** |  |  |  |  |  |  |  |  |  |  |  |  |  | 0.188 | 0.682** | 0.387 | 0.510** | - 0.218 | 0.476* | 0.417* | 0.118 | 0.418* |
|  | **p** |  |  |  |  |  |  |  |  |  |  |  |  |  | 0.367 | <0.001 | 0.056 | 0.009 | 0.296 | 0.016 | 0.038 | 0.573 | 0.038 |
| **Physical Discomfort** | **r** |  |  |  |  |  |  |  |  |  |  |  |  |  |  | 0.490* | 0.413* | 0.521** | - 0.349 | 0.267 | 0.256 | -0.200 | 0.318 |
|  | **p** |  |  |  |  |  |  |  |  |  |  |  |  |  |  | 0.013 | 0.040 | 0.008 | 0.087 | 0.198 | 0.217 | 0.337 | 0.122 |
| **BDI** | **r** |  |  |  |  |  |  |  |  |  |  |  |  |  |  |  | 0.557** | 0.695** | - 0.377 | 0.538** | 0.525** | 0.055 | 0.554** |
|  | **p** |  |  |  |  |  |  |  |  |  |  |  |  |  |  |  | 0.004 | <0.001 | 0.063 | 0.006 | 0.007 | 0.794 | 0.004 |
| **GDS** | **r** |  |  |  |  |  |  |  |  |  |  |  |  |  |  |  |  | 0.532** | - 0.269 | 0.246 | 0.188 | 0.310 | 0.428* |
|  | **p** |  |  |  |  |  |  |  |  |  |  |  |  |  |  |  |  | 0.006 | 0.194 | 0.236 | 0.367 | 0.131 | 0.033 |
| **STAI-T** | **r** |  |  |  |  |  |  |  |  |  |  |  |  |  |  |  |  |  | - 0.575** | 0.382 | 0.588** | 0.045 | 0.570** |
|  | **p** |  |  |  |  |  |  |  |  |  |  |  |  |  |  |  |  |  | 0.003 | 0.059 | 0.002 | 0.832 | 0.003 |
| **AES** | **r** |  |  |  |  |  |  |  |  |  |  |  |  |  |  |  |  |  |  | -0.117 | -0.144 | -0.030 | -0.385 |
|  | **p** |  |  |  |  |  |  |  |  |  |  |  |  |  |  |  |  |  |  | 0.577 | 0.491 | 0.887 | 0.058 |
| **ESS** | **r** |  |  |  |  |  |  |  |  |  |  |  |  |  |  |  |  |  |  |  | 0.278 | 0.120 | 0.378 |
|  | **p** |  |  |  |  |  |  |  |  |  |  |  |  |  |  |  |  |  |  |  | 0.179 | 0.567 | 0.062 |
| **RBDSQ** | **r** |  |  |  |  |  |  |  |  |  |  |  |  |  |  |  |  |  |  |  |  | 0.000 | 0.273 |
|  | **p** |  |  |  |  |  |  |  |  |  |  |  |  |  |  |  |  |  |  |  |  | 0.998 | 0.187 |
| **MoCA** | **r** |  |  |  |  |  |  |  |  |  |  |  |  |  |  |  |  |  |  |  |  |  | -0.256 |
|  | **p** |  |  |  |  |  |  |  |  |  |  |  |  |  |  |  |  |  |  |  |  |  | 0.217 |
| * p<0.05 **p<0.01  Abbreviations: r = Spearman’s rank correlation coefficient; CRST = Clinical Rating Scale for Tremor; MDS-UPDRS = Movement Disorder Society–Unified Parkinson’s Disease Rating Scale; ADL = Activities of daily living; NMSQuest = non-motor Symptoms Questionnaire; SF-36 = Short-Form-36 questionnaire; PCS = Physical Component Scale; MCS = Mental Component Scale; PDQ-39 = 39-item Parkinson’s Disease Questionnaire; BDI = Beck Depression Questionnaire; GDS = Geriatric Depression Scale; STAIT = State-Trait Anxiety Scale – “trait” anxiety; AES = Apathy evaluation scale; ESS = Epworth Sleepiness Scale; RBDSQ = REM Sleep Behavious Disorder Questionnaire; MoCA = Montreal Assessment of Cognition; FAQ = Functional Activities Questionnaire | | | | | | | | | | | | | | | | | | | | | | | |

**Suppl. Table S5: Characteristics of individual side effects after MRgFUS***

|  | **Pt1** | | | **Pt2** | | | **Pt3** | | | **Pt4** | | | **Pt5** | | | **Pt6** | | | **Pt7** | | | **Pt8** | | | **Pt9** | | | **Pt10** | | |
| --- | --- | --- | --- | --- | --- | --- | --- | --- | --- | --- | --- | --- | --- | --- | --- | --- | --- | --- | --- | --- | --- | --- | --- | --- | --- | --- | --- | --- | --- | --- |
| **T1** | P | mi | c | G | mi | c | U | mi | c | P  T | mi  mi | p  c | G  W^#^  Da | sev  sev  sev | p  p  c | - |  |  | P  G  W  Dm  Da | mi  mi  mo  mi  mo | c  c  p  c  p | G | mo | c | G | sev | c | G | mi | p |
| **T2** | - |  |  | - |  |  | I | mi | c | - |  |  | - |  |  | - |  |  | I | mo | p | - |  |  | - |  |  | I | mi | no |
| **T3** | - |  |  | - |  |  | n/a |  |  | - |  |  | - |  |  | - |  |  | - |  |  | - |  |  | - |  |  | - |  |  |
|  | **Pt11** | | | **Pt12** | | | **Pt13** | | | **Pt14** | | | **Pt15** | | | **Pt16** | | | **Pt17** | | | **Pt18** | | | **Pt19** | | | **Pt20** | | |
| **T1** | U | mi | c | P  U | mi  mi | no  c | P  T  G | mi  mo  mi | c  p  c | T  U | mi  mi | c  c | G | mo | p | G  W | mo  mo | c  c | - |  |  | P  G  W | mi  mi  mi | c  p  c | U | mi | p | P  G  W  Dm  Da | mo  sev  mo  mi  mi | p  p  p  c  c |
| **T2** | - |  |  | T | mo | no | I | mo | no | - |  |  | - |  |  | T | mi | no | - |  |  | - |  |  | - |  |  | - |  |  |
| **T3** | - |  |  | - |  |  | - |  |  | - |  |  | - |  |  | - |  |  | n/a |  |  | - |  |  | n/a |  |  | n/a |  |  |
|  | **Pt21** | | | **Pt22** | | | **Pt23** | | | **Pt24** | | | **Pt25** | | |  | | |  | | |  | | |  | | |  | | |
| **T1** | P  T  G | mi  mi  mi | no  no  c | G | mi | c | G  Dm | mi  mi | c  no | G  W  Dm | mo  mo  mo | p  p  c | P^#^  T  G  W | mi  mo  mi  mi | c  p  c  c |  |  |  |  |  |  |  |  |  |  |  |  |  |  |  |
| **T2** | - |  |  | - |  |  | - |  |  | I | mi | p | - |  |  |  |  |  |  |  |  |  |  |  |  |  |  |  |  |  |
| **T3** | - |  |  | n/a |  |  | - |  |  | n/a |  |  | - |  |  |  |  |  |  |  |  |  |  |  |  |  |  |  |  |  |
| Pt = Patient, T1 = 1-3 days post, T2 = 6 months post, T3 = 12 months post MRgFUS, Da = Dysarthria, Dm = Dysmetria, G = objective gait ataxia, I = involuntary movements (subjective or observed on physical examination), P = paraesthesia, U = subjective unsteadiness, T = taste disturbances, W = weakness, mi = mild, mo = moderate, sev = severe, c = completely regredient, p = partially regredient, no = not regredient, n/a = not available (no further follow up)  * The first column indicates the side effect, the second the severity (rated as mild, moderate or severe) and the third the regredience of side effects (rated as not, partially or completely regredient)  ^#^ Weakness and paraesthesias in multiple body parts were combined in “W” and “P” | | | | | | | | | | | | | | | | | | | | | | | | | | | | | | |

**
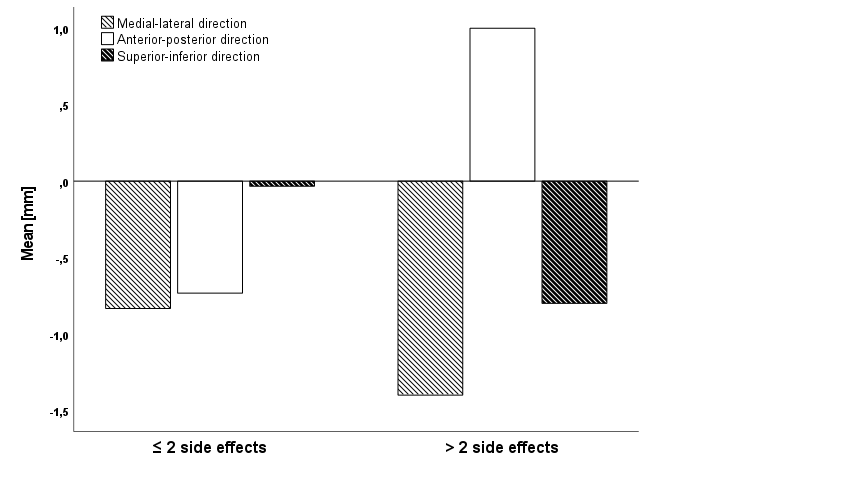
**

**Suppl. Figure S1: Mean target adjustments in relation to the occurrence of side effects**. In patients with more than 2 side effects target adjustments were made with respect to the standard coordinate mainly in lateral, anterior and superior direction.
